# Controlled Synthesis and Microstructural Properties of Sol-Gel TiO_2_ Nanoparticles for Photocatalytic Cement Composites

**DOI:** 10.3390/nano9010026

**Published:** 2018-12-25

**Authors:** Elena Cerro-Prada, Sara García-Salgado, M. Ángeles Quijano, Fernando Varela

**Affiliations:** 1 Departamento de Construcción, Infraestructura y Transporte, Escuela Técnica Superior de Ingeniería Civil, Alfonso XII, 3, Universidad Politécnica de Madrid, 28014 Madrid, Spain; fernando.varela@upm.es; 2 Departamento de Hidráulica y Ordenación del Territorio, Escuela Técnica Superior de Ingeniería Civil, Alfonso XII, 3, Universidad Politécnica de Madrid, 28014 Madrid, Spain; sara.garcia@upm.es (S.G.-S.); marian.quijano@upm.es (M.Á.Q.)

**Keywords:** TiO_2_ photocatalysis, anatase, cement-based composites, microstructural characterization, TiO_2_ nanoparticles, sol-gel synthesis

## Abstract

Titania nanoparticles are intensely studied for photodegradation applications. Control of nanoscale morphology and microstructural properties of these materials is critical for photocatalytic performance. Uniform anatase-type TiO_2_ nanoparticles were prepared by the sol-gel process using titanium isopropoxide as precursor. Controlled annealing up to 400 °C established crystallization and particle size ranging between 20 and 30 nm. Detailed thermal examination reveals that anatase phase transformation into rutile is affected by the annealing temperature and by the initial particle size. The anatase to rutile phase transformation occurs in the nanoparticles at 550 °C. The Total Reflection X-ray Fluorescence (TXRF) study of the anatase nanoparticles shows a shift towards higher energy in the Ka Ti line of 10 eV, related to structural defects. These features were discussed in the photocatalytic behavior of several cement-based materials modified with the so-prepared anatase nanoparticles. The photocatalytic activity of the anatase-type TiO_2_/cement mortar system is evaluated from the degradation of Methylene Blue (MB) under UV irradiation, monitored through the absorbance at 665 nm. The results show that the photocatalytic composites exhibit up to 76.6% degradation efficiency. Mechanical testing of the nano-TiO_2_ modified cementitious composites evinces a moderate reinforcement of the strength properties at long ages.

## 1. Introduction

The cement industry is facing great concerns due to the fact that its activity has been identified as one of the main sources of air pollution. Along with emerging strategies focused on developing alternative fuels to reduce the important greenhouse effect of the emissions, the scientific community is broadly addressing the use of innovative cements made of a suitable combination of “green binders”, such as metakaolin and nano-silica [1]. Other efforts to mitigate the environmental impact of cement production are based on the incorporation of nanomaterials, which have been proven to provide interesting solutions for pollution remediation. In this sense, heterogeneous photocatalytic degradation of organic contaminants over nanotitania has impulsed the development of photocatalytic cement, which is in the market since decades. However, such materials are no still intensively used mainly due to the light intensity requirements to activate the titanium dioxide’s photocatalytic properties. Therefore, further research should be conducted in order to identify which features for the nanocatalysts are required to provide efficient photocatalytic performance.

For efficient accommodation of such new construction materials, the nanosized component should be fundamentally studied in order to address its complementarity and performance. Photocatalytic cement-based materials are a relatively new group of smart materials produced by addition of photo-activated heterogeneous semiconductors in the form of nanoparticles [2,3,4]. These composites exhibit photocatalytic properties making self-cleaning applications possible [5,6,7]. Furthermore, photocatalytic cement allows heterogeneous photocatalysis processes in certain organic species which results in the oxidative mineralization of pollutants [8,9,10].

In the past decade, concrete and cement mortar containing nano-sized titanium dioxide have been found capable of degrading organic compounds for waste water treatment technologies [11] and urban air decontamination [12]. Titanium dioxide in anatase form is almost without exception, the most featured and widely used semiconductor for commercial photocatalytic applications [13]. The photocatalytic performance of titania has been found to be even more efficient if the semiconductor is in nanoparticle form [14], due to its high specific surface area to volume ratio, which enables improvements in catalysis. Besides, a cement matrix provides an adequate support with porous microstructure and large internal specific surface area, which enhances the photocatalytic activity [15]. Additionally, titania nanoparticles have unique physical properties that can add smart functionality to cementitious materials beyond the photocatalytic properties. Hence, there is a synergistic effect between nano-TiO_2_ and cement that suggests an emergent self-assembly route for functionalized cement engineering [16].

Many attempts have been made in recent years to provide new strategies in the manufacture of photocatalytic cement. Relevant research has confirmed the need to continue studying the influence of different additions on the photocatalytic action, the type of catalyst added to the building material and the method of preparation of titania dioxide in the form of a nanoparticle. Cárdenas et al. [17] conducted a study of photocatalytic activity in white Portland cement pastes (IM CEM) added with anatase and rutile nanoparticles. They used different addition percentages of TiO_2_ (0.5%, 1.0% and 3.0%) as well as different anatase to rutile ratios (100:0, 85:15, 50:50). The samples consisted of cement paste discs of 2.8 cm diameter and 0.5 cm thickness prepared with a water to cement ratio of 0.5. The results showed that at early ages the samples with the highest photocatalytic activity corresponded to those of 3% in anatase phase, whereas at late ages those corresponding to 3% 50:50 anatase to rutile, highlighting the importance of the crystalline phase of the catalyst. Lucas et al. [18] inserted low concentrations of TiO_2_ nanoparticles in cement mortars. The authors used commercial nano-TiO_2_ (Degussa P25) with a ratio of 85% of anatase and 15% rutile. For low concentrations of nano-TiO_2_ (0, 0.5, 1, 2.5 and 5 wt.% of total solid content), the authors obtained NOx degradation rates close to 80% with only 1 wt.% TiO_2_. These investigations are interesting examples among numerous works focused on exploring the possibilities of improving photocatalytic efficiency in cement-based materials with low additions of titanium dioxide nanoparticles.

However, one of the fundamental concern of the pollution-degradation technology is to ensure that the cementitious material retains, at least, its structural capacity after modifying its microstructure with the addition of photocatalytic nanoparticles. In this sense, many authors have reported that the addition of metal oxide nanoparticles accelerates chemical reactions during initial hydration, thus reinforcing cement compounds [19]. The metal oxide nanoparticles react with CaOH increasing the amount of calcium silicate hydrated (CSH) produced, giving rise to a more compact microstructure, which not only decreases the permeability, but also improves the mechanical properties such as compressive strength, flexural and abrasion. Thus, Meng et al [20] carried out studies on cement mortars with additions of nano-TiO_2_ and found that the presence of the nanomaterial increases the resistance to compression of high strength mortars up to 15.8%, obtaining the maximum resistance gain when adding 2% cement weight nanomaterial content. Also, the previously mentioned authors Lucas et al., reported mechanical properties of cement mortars modified with low concentrations of nano-TiO_2_. Their results showed that, after 28 days of hydration, the compression resistance slightly increases for concentrations of TiO_2_ equal to or less than 1%, while the strength decreases for higher TiO_2_ concentrations.

Nevertheless, there is still a great controversy among researchers about how the inclusion of nanoparticles in the cement matrix affects the mechanical performance and durability of the material. It is clear that, in order to increase the nucleation and growth of the CSH gel, the nanoparticles must promote the pozzolanic reaction, either by the siliceous nature of the nanomaterial (as in the case of SiO_2_) or by causing microstructure densification, inhibiting the growth of Ca(OH)_2_ crystals in favor of an increased production of CSH gel. The latter is, we believe, the most likely effect that TiO_2_ nanoparticles cause on the cementitious material.

Concerning the TiO_2_ nanoparticle preparation method, there exist several ways to synthesize nano-TiO_2_; however, at the industrial level, three methods predominate. Sulphate method [21] allows obtaining titanium oxisulfate through a solution of titanium-containing minerals, such as ilmenite (FeTiO_3_). In sulfuric acid, ilmenite is neutralized with a base, giving rise to a hydrous oxide of titanium (TiO_2_·nH_2_O), which is calcined to form TiO_2_ in the form of anatase or rutile. The second method, known as chloride method or chemical vapor deposition method [22], consists of the thermal decomposition or combustion of titanium tetrachloride vapor, which is formed by the reaction of titanium minerals and chlorine gas at temperatures between 973 and 1273 K. Titanium tetrachloride is then annealed producing TiO_2_ as a product. The third method is that of alkoxides [23], which are dissolved in solvents such as alcohol, and then mixed with another solution containing water, which hydrolyzes the alkoxide to result in a TiO_2_ sol. Finally the sol is gelled to form hydrated TiO_2_ and then calcined to form titanium dioxide. The so-called sol-gel method allows to produce high purity materials in powder-type, thin films, fibers, monoliths and self-supported bulk structures. In addition, this method has several advantages over other synthesis techniques, such as purity, homogeneity and stoichiometric control, easy preparation under ambient conditions, low synthesis temperature and handling of particle size, shape and distribution.

Because the photocatalytic reactions of interest occur at surfaces, small particle size is desirable to maximize surface area. Hence, an improvement in the controlled synthesis of titania nanoparticles is required in order to exploit their properties in applications, as not only the chemical composition and the physical arrangement of the atoms but also the particle size influences the behavior of this material. Among the most extended methods to fabricate TiO_2_ nanoparticles, sol-gel method has been extensively used [24,25,26,27,28] offering good results. However, one commonly encountered problem is that sol-gel like methods may generate amorphous or low crystallinity products, which necessitates a subsequent annealing for crystallization or further crystallization. Such a thermal annealing may cause hard aggregation and sintering, as a collective result of thermally activated inter-particle adhesion [29]. Additionally, because the anatase phase was shown to have higher photocatalytic activity than rutile, phase stability as a function of particle size is relevant.

To address the above issues, sol-gel synthesis followed by controlled annealing is employed in this investigation. Our method ensures high crystallinity in the final material and allows for a high control of the particle size. Anatase sols were subjected to microstructural, morphological, and compositional study. The as-prepared crystalline nanoparticles were incorporated into bulk cement mortar to determine mechanical properties and photocatalytic efficiency of the anatase sols cement based composites.

In summary, major contributions of this work are that (1) highly crystalline TiO_2_ anatase nanoparticles are prepared by sol-gel followed by controlled annealing and (2) photocatalytic cement-based material can be prepared by incorporating such anatase nanoparticles in traditional mortar and no detriment to long-term mechanical properties is found.

## 2. Results

### 2.1. TiO_2_ Nanoparticles Characterization

#### 2.1.1. X-ray Diffraction (XRD) Studies

**Figure 1:**
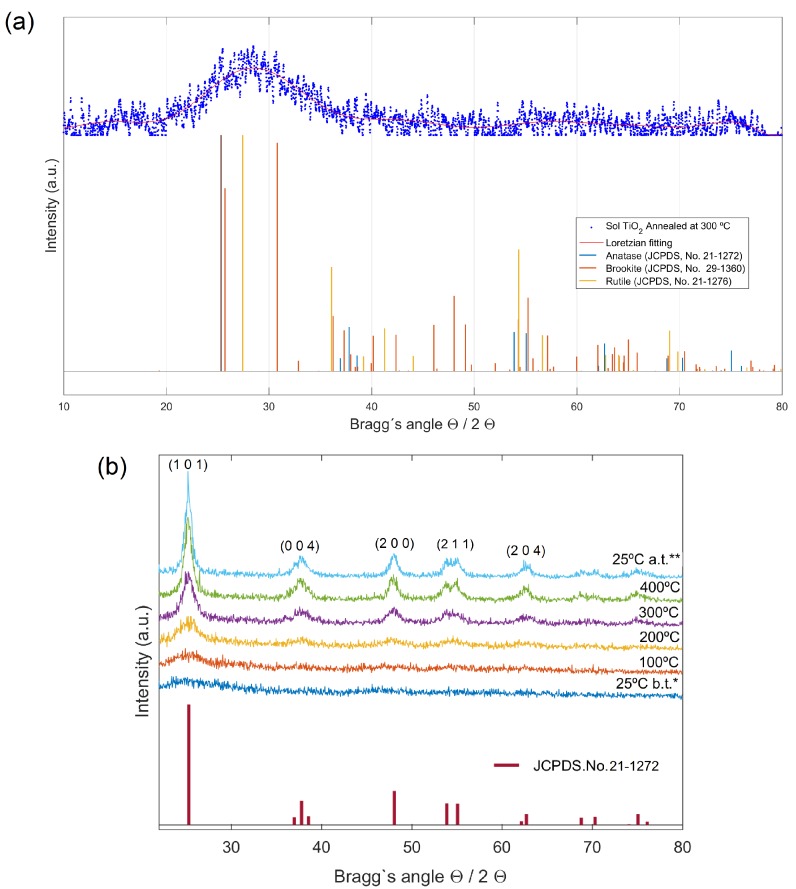
(**a**) XRD spectra of nano-TiO_2_ samples prepared by sol-gel synthesis at ambient temperature followed by calcination at 300 °C. XRD data was fitted with Gaussian–Lorentzian function (G/L = 30) and variable FWHM. Despite poor crystallinity, multiple phases were detected in these samples. (**b**) XRD patterns of as prepared sol-gel nano-TiO_2_ sample during the heating period from 25 to 400°, recorded at different temperatures (*25 °C b.t. (before thermal treatment), 100 °C, 200 °C, 300 °C, 400 °C and **25 °C a.t. (after treatment). The presence of crystalline anatase is identified in the samples heat-treated from 200 up to 400 °C.

XRD data of the sol-gel nanotitania powders, heat-treated between 100 and 400 °C at the rate of 1.5° $\min^{-1}$, were taken and shown in Figure 1. No crystallinity was observed in XRD spectra of samples annealed at 100 and 200 °C. Samples annealed at 300 °C, however, featured low crystallinity (17.9%), with scarce presence of anatase, rutile and brookite (Figure 1a). The presence of the three different crystalline forms of TiO_2_, should not be surprising since samples synthesized at low temperatures are often scarcely crystalline and the corrected ratio could be easily reached by calcination at relatively high temperatures [30].

In order to study the phase transitions and crystallite growth that take place during the annealing process, X-ray diffraction at variable temperature conditions was performed in the nano-TiO_2_ samples prepared by sol-gel at ambient temperature with no further calcination. Shown in Figure 1b, is the diffraction data of a single as-prepared sol nano-TiO_2_ sample, measured on the Siemens diffractometer at 25 °C (before annealing), 100 °C, 200 °C, 300 °C, 400 °C and 25 °C (after annealing is completed). As observed in Figure 1b, when the sample is heat-treated up to 400 °C, clearly displays the presence of crystalline phases in preliminary agreement with anatase (JCPDS.No.21-1272). Although anatase is the phase normally found in sol-gel synthesized of TiO_2_, brookite is often observed as a by-product when the precipitation is carried out in an acidic medium at low temperature [31]. It should be accounted that the main (101) diffraction peak of anatase at 2Θ=25.28° overlaps with the (120) and (111) peaks of brookite at 2Θ=25.34° and 25.69°, respectively, so that apparently pure anatase or brookite samples can be a mixture of anatase and brookite. To assess the existence of brookite in the samples, we used one characteristic peak of anatase at 2Θ=62.57°, which does not overlap with any diffraction peak of brookite. The aforementioned anatase peak was detected with 12.7% relative intensity in sample tested at 25° after annealed at 400 °C, in agreement with the corresponding JCPDS data set. In the other hand, the second main (121) diffraction peak of brookite at 2Θ=30.80°, was not found in the diffractograms, which indicates that brookite phase was not present in such samples. Therefore, these results clearly accomplish that: (i) highly pure anatase phase has been obtained, and (ii) there exists a dependence of the crystallization rate of sol-gel anatase with temperature, achieving its full crystallization at 400 °C.

The crystallite size (*d*) for the prepared samples was determined by measuring the broadening of a most intense peak of the phase (main peak) in a diffraction pattern associated with a certain planar reflection within the crystal unit cell according to the Debye−Scherrer equation using the measured full width at half-maximum (FWHM) values of the XRD peaks. A correction factor of k=0.9 was used for particle shapes. The FWHM value for the peak of maximum intensity was chosen to be (101) reflection. Typical values of crystallite sizes have been calculated to be 8.2±0.1 nm for the sol-gel synthesized TiO_2_ nanoparticles annealed at 400 °C. The average crystallite size gradually increased from 6 to 8 nm with an increase of temperature from 200 up to 400 °C, reaching its final value of about 9 nm after cooling from 400 °C to ambient temperature, in agreement with reported values for sol-gel synthesized TiO_2_ ([22]).

#### 2.1.2. Morphology

**Figure 2:**
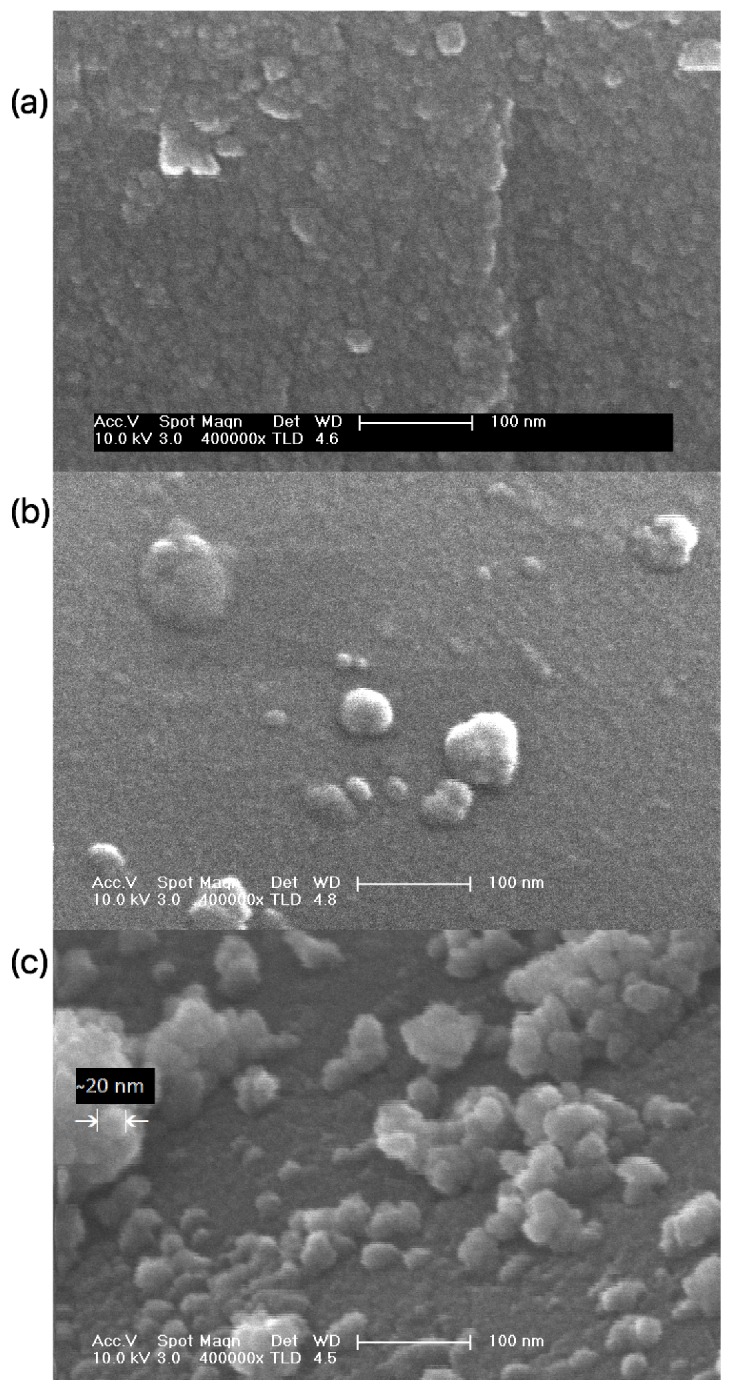
SEM micrographs of sol-gel nano-TiO_2_ calcined at different temperatures: (**a**) annealed at 100 °C, (**b**) 300 °C, (**c**) 400 °C. It can be observed that annealing can improve crystallinity of the nanoparticles through promoting grain growth and recrystallization.

Figure 2 displays the SEM micrographs of sol-gel TiO_2_ nanoparticles heat-treated at different temperatures. As shown in Figure 2a, the sample calcined at 100 °C consists of particles with spherical morphology and poor aggregation, which only takes place during the crystal growth process at higher temperatures, i.e., 300 and 400 °C (Figure 2b,c). It should be noticed that, the higher annealing temperature, the larger aggregate size with spherical morphology is obtained. For the sample calcined at 400 °C (Figure 2c), the grain boundaries are clearly observed in the SEM images. Because of the poor contrast in the micrographs, it is difficult to exactly measure the size of the primary spherical particles accurately. By using ImageJ software on the micrograph shown in Figure 2c, average nanoparticle diameter is estimated to be about 20–30 nm, although we would like to point out that the particle size determination by means of SEM analysis is not accurate. The nanoparticles prepared by annealing at 400 °C showed some non-uniformly distributed agglomerations consisting of small particles and displayed a broad particle size distribution.

#### 2.1.3. Total Reflection X-Ray Fluorescence Analysis

**Figure 3:**
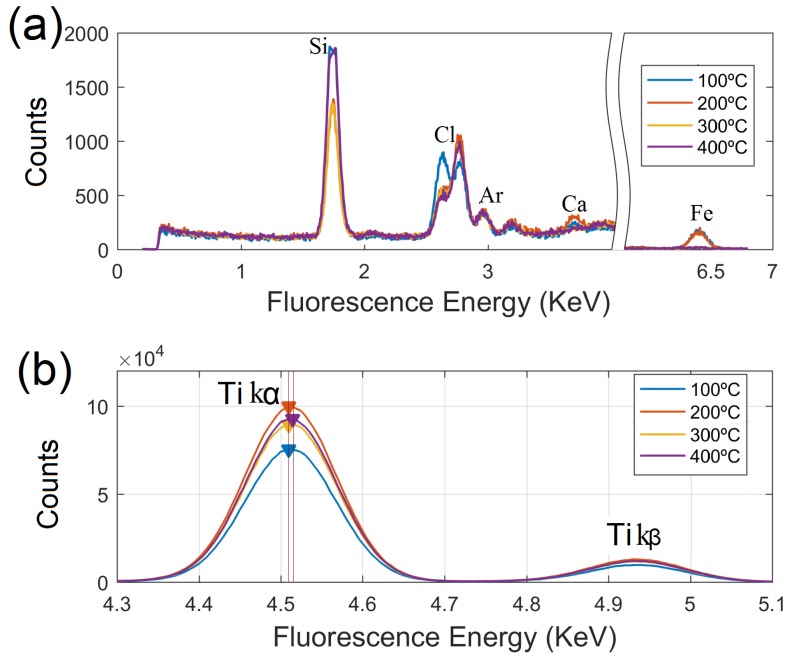
TRXF spectra of nano-TiO_2_ samples prepared by sol-gel synthesis at ambient temperature followed by calcination up to 400 °C. (**a**,**b**) Ti Kα and Kβ fluorescence lines in the samples.

The samples were analyzed with the TRXF system just after their sol-gel preparation. The fluorescence spectra were split in two regions as shown in Figure 3. In the first panel, Figure 3a, the energy range from 4.3 to 5.1 KeV has been removed from the plots, displaying the peaks that emerge with low intensity. Due to the silicon nature of the carrier used in the experiments, strong Si peaks were presented in the spectra. Low contents (close to the limit of detection) of Cl, Ar, Ca and Fe were observed in the samples. The present of Ar was clearly expected due to the absorption effects of light Z elements when TRXF measurements are performed in air conditions [32]. Cl can be identified as trace constituent in the samples, as its strongest principal peaks (Kα and Kβ) were found in the spectra with decreasing net intensity when annealing temperature increases. The presence of Cl in the samples is most probably due to the acidic aqueous conditions under which nano-TiO_2_ sols were prepared. The sol-gel preparation method ends up with some Cl ions remaining on the surface of the samples; such impurities, however, are easy removed after the thermal treatment is applied. Weak peaks of Ca and Fe were revealed in the spectra of the samples heat treated up to 200 °C. These impurities were probably arising from the background of TRXF test or the residual precursors. At higher temperatures, both Ca and Fe signals were markedly attenuated and therefore, did not reach significance in the samples annealed at 300 and 400 °C.

The region of interest for studying the Kα and Kβ peaks of titanium, can be located from 4.3 to 5.1 KeV within the fluorescence spectra, as shown in Figure 3b. The strongest Kα fluorescence line results from 2p to 1s transitions, approximately eight times weaker is the 3p-1s transition (Kβ). A loss of intensity is observed in the Kα line for the sample annealed at the lowest temperature (100 °C), which is indicative of partial particle oxidation.

A more remarkable feature is revealed in the sample heat treated at (400 °C), whose Kα Ti line slightly shifted towards higher energy with Δ$E_{F}$ by ≈10 eV, and could be linked to a dominant presence of structural defects at the particle surface [33].

#### 2.1.4. Microstructural Evolution during Annealing

**Figure 4:**
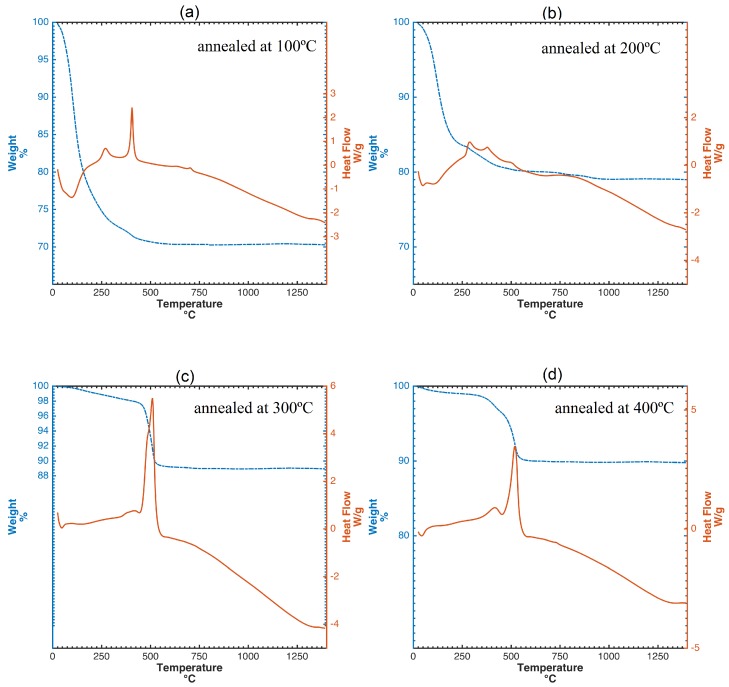
TGA and DSC curves of nano-TiO_2_ sols calcined at different temperatures: (**a**) annealed at 100 °C, (**b**) 200 °C, (**c**) 300 °C, (**d**) 400 °C. (**a**–**c**) Anatase phase transformation into rutile is affected by the annealing temperature and (**d**) by the initial particle size.

The evolution of the crystal structure during the annealing process can be observed by differential scanning calorimetry (DSC) curves (Figure 4). This study confirms the well known fact that the thermal behavior of TiO_2_ usually depends on the chemical composition, preparation conditions and existing phases. Figure 4a shows the thermogravimetric analysis (TGA) and DSC curves of the sol-gel nano-TiO_2_ samples calcined at 100 °C. There are three features in these curves. The first weight loss of ca. 24%, in the temperature range of 50–245 °C, is attributed to the loss of small molecular compounds, such as water and ethanol in the xerogel texture, corresponding to a gradual endothermic process from the DSC curve. More precisely, the endothermic peak at 99 °C with mass loss of 13.78% was due to the desorption of physically adsorbed water. The decomposition of organic species also gives rise to a smooth exothermic peak at 322 °C in the DSC curve. The third weight loss of ca. 12.5% in the range of 360–480 °C is, again, attributed to the gradual removal of the organic residues. Finally, The exothermic peak at approximately 393 °C did not show any weight loss and should be the phase transformation from amorphous TiO_2_ to anatase. Both TGA and DSC curves show little change in the range of 480–1400 °C. This denotes that the residues have been removed and a stable phase structure has been formed.

In Figure 4b, the TGA and DSC curves of the nano-TiO_2_ sols annealed at 200 °C are presented. Endothermic phenomena related to crystallization were not observed in the DSC curves. Three small exothermic peaks at 250 –400 °C were due to the decomposition and oxidation of organic substances as well as the transformation of TiO_2_ from amorphous to anatase phase with poor crystallinity. TGA/DSC curves of nanotitania sols calcined at 300 and 400 °C, respectively, are shown in in Figure 4c,d. Both curves show similar trends. Specifically, sol-gel titania samples annealed at 400 °C, feature an exothermic effect with a smooth peak occurring at temperature close to 600 °C. These samples were identified as singular anatase phase by the XRD characterization, which implies that only anatase transforms into rutile during the heating process. The transition temperature of this transformation is in the range between 350 and 1175 °C, which in agreement with [34]. Untypically, anatase transformation into rutile occurs above 800 °C [35], as a consequence of the thermodynamic stability and the activation energy dependence of the crystallite size. Therefore, if anatase is thermodynamically stable, the phase transition does not begin when heating until the critical particle size and the activation energy are reached. In the initial stage, sol-gel anatase nanoparticles annealed at 400 °C displayed a 15.4 nm crystallite size, as measured by Debye–Scherrer formula, which is not in its stability zone <11 nm [36]. Thus, this thermodynamic instability contributes to a higher transition temperature.

#### 2.1.5. Mechanical Properties of Nano TiO_2_ Sols-Mortar

**Figure 5:**
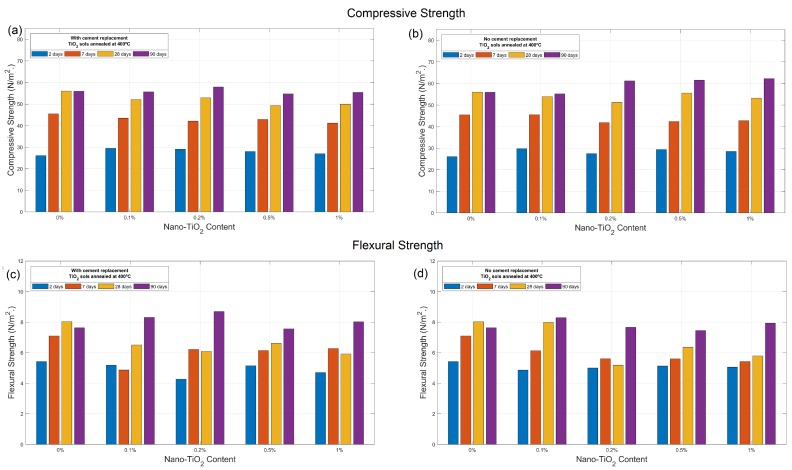
(**a**,**b**) Compressive and (**c**,**d**) flexural strength of cement composites containing nano-TiO_2_ sols annealed at 400 °C, with and without cement replacement.

Table 1 presents the compressive and flexural strengths for cement mortars with nano-TiO_2_ sols annealed at 400°, with and without cement replacement, for all test ages (1, 7, 28 and 90 days). Figure 5 also shows the trends of compressive and flexural strength by varying the amount of TiO_2_ content. For samples prepared with cement replacement, at early and middle ages of mortar (2, 7 and 28), slightly decreased strength is obtained for a replacement content of nano-TiO_2_ of 0.1%, 0.5% and 1%. By replacing the TiO_2_ in cement with a 0.2%, however, slight improvement is obtained in both compressive (3.6%) and flexural strength (13.7%) at long ages. In the case of mortar prepared with addition of TiO_2_ with no cement replacement, as presented in Figure 5b,d, enhanced compression behavior is obtained at long ages for TiO_2_ content 0.2%, 0.5% and 1% by weight of cement, reaching compressive strength improvements up to 11.4% at long ages. However, this behavior cannot be clearly observed for flexural strength, indicating heterogeneity in the dispersion of the nanoparticles within the cementitious matrix.

Although this research does not attempt to fix the optimal content of TiO_2_ nanoparticles to produce remarkable improvements in the mechanical behavior of the cement mortar, these results confirm that the presence of sol-gel Ti nanostructures in the cementitious material induces the densification of the microstructure through a filling effect that does not alter the structural capacity of the material, favoring the process of setting and improving the ultimate durability of the material, and showing the validity of this self-assembly route for functionalized cement engineering.

#### 2.1.6. Photocatalytic Efficiency

**Figure 6:**
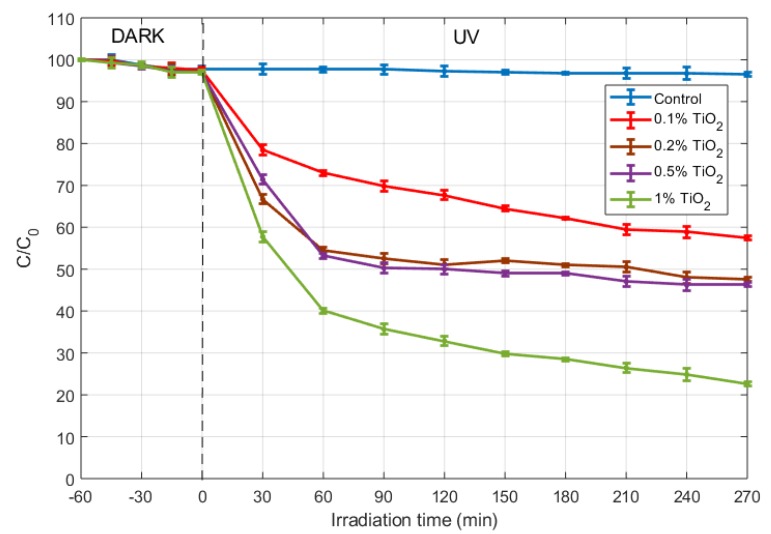
Photocatalytic MB dye degradation percentage with various time intervals for anatase sols inserted in mortar with different TiO_2_ content.

The graph in Figure 6 shows the concentration changes of methylene blue dye (MB). An adsorption period of 60 min in the dark was observed. During that period, the rate of disappearance of MB was found to be equal to $6.5\times 10^{-3}\;\mu$ mol $l^{-1}$ $\min^{-1}$, hich produces a total 2.1% degradation rate. At the end of that period, the initial MB concentration for the samples was 17.52±0.2. Afterwards, the anatase sols inserted in mortar revealed photocatalytic efficiency under the irradiation of UV lamp. It was found that MB degradation ratio were 41.03%, 50.97%, 52.34% and 76.60% after 270 min for 0.1%, 0.2%, 0.5% and 1% TiO_2_ content, respectively.

The authors have already reported photocatalytic studies on nano-TiO_2_-cementitious materials, specifically cement paste with addition of nano sol-gel TiO_2_ [16]. In such study, the same sol-gel procedure was used to synthesizing the titania nanoparticles, although the photocatalytic sols had none or poor crystallinity because controlled annealing was then not performed, as that is now the aim of this work. It can be seen that those previous results (with amorphous TiO_2_) are now doubled for anatase-type TiO_2_ inserted in mortar with 1% TiO_2_ concentration. These findings show that the increase of the photocatalytic performance is of a factor close to 2 after crystallization [37]. Moreover, the obtained results are consistent with Rtimi [38], who reported similar trends for TiO_2_ concentrations of 0.1% by weight, although in our study the porosity of the substrate (cement matrix) induces a slightly higher photocatalytic efficiency. According with Kadirova [39], suitable supports for TiO_2_ to catalyze the photodegradation process, should meet the following requirements:

- the support must be resistant to oxidative radicals generated in aqueous solution and have high surface area, an accessible volume of micropores, and narrow pore size distribution.
- the photodegradation of organic pollutants must proceed with acceptable kinetics.

Cement mortar microstructure fully meets the first criterion, and our results show that an acceptable kinetic is obtained for the photodegradation of MB in the presence of anatase sols inserted in cement mortar.

## 3. Discussion

This paper focuses on two issues considered to be key in the photocatalytic performance of TiO_2_ catalysts supported in cement-base matrix: (i) a controlled sol-gel route is proposed to produce anatase nanoparticles with small particle size and (ii) Mortar microstructure provides adequate support for the photocatalytic action of anatase nanoparticles, which do not alter the structural properties of the cementitious material.

A nanocrystalline anatase powder has been prepared by the sol-gel method through hydrolysis of titanium-isopropoxide alcoholic solution and then controlled calcination of the resultant suspension up to 400°. According to DRX results and particle size estimation by the Debye–Scherrer equation, the sol-gel nano-TiO_2_ heat treated at 400° consists of anatase 8 nm size crystallites, with diameter of nanoparticles of ≈20 nm. Nanoparticle morphology is observed to be almost spherical, which is due to acidic condition that promotes agglomeration. The examination of TRXF tests indicate the presence of structural defects on the surface of the nanoparticles after the annealing process at 400 °C. TGA analysis reveal the transformations from amorphous TiO_2_ to anatase and then from anatase to rutile, highlighting that anatase phase transformation into rutile is affected by the annealing temperature and by the initial particle size. Mechanical performance results of mortars with inclusions of anatase sols do not indicate that the structural properties of the construction material are significantly altered. In fact, a certain increase in the compression strength at long ages is observed, probably due to a densifying and filling effect that TiO_2_ nanoparticles promote in the cement matrix.

Detailed studies of photocatalytic degradation of MB dye through nano anatase cement mortar composites, shows a degradation of 76.6% after 270 min of irradiation when 1% of cement weight anatase sols content is added to mortar, which establishes a efficient route for producing TiO_2_ cement composites for photocatalytic applications.

## 4. Materials and Methods

### 4.1. Sample Preparation

The nano-TiO_2_ sols were prepared by hydrolysis and condensation reaction of titanium tetraisopropoxide (TTIP, Aldrich, 97%) dissolved in dry ethanol (Aldrich, 99.9%), at a percentage of 23.8% and 76.2% respectively. The resulting solution was partially hydrolyzed by adding a mixture of distilled water, chloride acid, and ethanol (0.48%, 0.92% and 98.6%, respectively). The TTIP/H_2_O/HCl molar ratio was 1:4:0.04. Then, the mixture was dried for 24 h at ambient temperature. The obtained solid material was then divided into four samples which were taken in a porcelain crucible and heated in a furnace at 100 °C, 200, 300 and 400 °C respectively, under ramp-type heating conditions detailed in Table 2. The samples were kept at the desired temperature during 2–10 min for stabilization, and left to cool for 8 h inside the switched-off furnace. At the end of the process, gray colored powders were obtained.

Mortar mixtures of CEM I 42.5-R Portland cement (Portland Valderrivas, Spain) were prepared with a water-cement or w:c ratio of 0.5. The aggregate in mortar was a siliceous–calcareous sand (0/4 mm). Prior to being mixed and allowed to react with water, cement clinker was combined with different concentrations of sol-gel titania nanoparticles (TiO_2_ contents in the mortar specimens were 0, 0.1, 0.2, 0.5 and 1 by weight of cement), with and without cement replacement, as described in Table 2 (mortar mix proportions per 768 cm^3^. Mortar samples were then cast in the form of prismatic (40 ×40× 160 mm) by pouring the slurry into plastic molds, sealed and afterward submerged in water. Finally, the hardened mortar was removed from the molds after 24 h and allowed to cure according to the curing conditions. Two mortar mixtures were prepared for each nano-TiO_2_ concentration, thus, six mortar samples for each concentration were tested.

### 4.2. Characterization

X-ray diffraction (XRD) was performed on the nano-TiO_2_ samples in a Siemens diffractometer using a Θ/2Θ configuration with 0.02° scan step and 6 s integration time. The phase identification was first obtained by using the Match! Software version 3.1.1 together with JCPDS database. Phase transformation and crystallite growth were studied as a function of temperature (25–400 °C) by collecting X-ray diffraction data on the diffractometer at selected temperatures (25, 100, 200, 300 and 400 °C).

Particle size and morphology of the titania nanoparticles were analyzed with Scanning Electron Microscope SEM Philips XL-30 S-FEG, equipped with W source detectors of secondary and backscattering electrons and a vacuum sample chamber with a vacuum of lower than $4\times 10^{-4}$ Pa. Prior to imaging, the samples were sputtered with a 12 nm thin film of chrome.

Total Reflection X-ray Fluorescence (TRXF) technique was used to determine the elemental concentrations of major, minor and trace elements in the annealed nanotitania sols samples and to examine the effectiveness of the sol-gel preparation method. Surface characterization of the so-formed TiO_2_ nanoparticles was also carried out by careful examination of the fluorescence spectra. The TRXF measurements were made using an S2 PICOFOX TRXF spectrometer (Bruker, Germany), equipped with a 50 kW Mo/W dual target X-ray tube and a Ni/C multilayer, 17.5 keV with 80% reflectivity. A Si(Li) detector with an active area of 10 mm^2^ and a resolution of 160 eV at 5.9 keV (Mn Kα) was used for detection and measurement of X-rays produced. Mo Kα excitation condition was set by means of the adequate arrangement of the monochromator geometry.

Thermal decomposition data and thermal behavior of the nanotitania samples were obtained by a TGA-DSC-DTA SDT Q600 Simultaneous Thermal Analyzer from TA Instruments. Samples (±20 mg) were placed in an alumina crucible and heated up to 1400 °C at a scan rate of 5 °C $\min^{-1}$. Continuous recordings of sample temperature, sample weight, its first derivative and heat flow were taken. An air-flow, with a flow rate of 20 cm^3^ $\min^{-1}$, was used to remove gaseous decomposition products.

The prismatic specimens of nanotitania-mortar composites were tested to determine the average compressive and flexural strength. The samples were loaded using an HM-S type frame universal testing machine, with 300 kN capacity, at the rate of 150 mm/min until failure, according to ASTM C349.

### 4.3. Photocatalytic Studies

The photocatalytic efficiency of sol-gel anatase nanoparticles for functionalized cement composites, is evaluated by measuring the degradation of MB under UV light illumination, monitoring through the absorbance at 665 nm. Cement mortar with 0.5 water:cement ratio, was mixed with anatase sols as prepared in previous section (Sample ID 400TiO_2_). TiO_2_ contents in the mortar specimens were 0, 0.1, 0.2, 0.5 and 1 by weight of cement. Cement class was CEM I 42.5-R. The aggregate in mortar was a siliceous–calcareous sand (0/4 mm). The following Table 3 resumes the mix proportions of the specimens (per 768 cm^3^):

A rotary mixer with a flat beater was used for mixing. First, sol-gel TiO_2_ nanoparticles were dissolved in water and stirred at high speed for about 2 min. Then cement clinker was added in the mixer and stirred for other 2 min. Afterwards, sand was added into the mixture and auto-stirred for about 1.5 min. For each well-mixed mortar mixture, three specimens were prepared by pouring the mix into molds of size 40×40× 160 mm. An external vibrator was used to facilitate compaction and decrease the amount of air bubbles. The samples were demolded after 24 h and then cured in air at room temperature for 2 days. To measure photocatalytic activity, samples with a thickness of 10 mm were obtained by cutting the mortar specimens into slices with a diamond saw cutter. Ten samples were used for each TiO_2_ concentration.

In a photocatalytic activity experiment, each mortar slice was immersed into 100 ml of methylene blue (Aldrich) solution ($18\times 10^{-6}$ M), and kept at dark for 1 h before irradiation, during which MB aliquots were taken out at time intervals to ensure that adsorption-desorption equilibrium was reached. Then, the specimen is taken to UV lamp (15 W, 365 nm) during the function of time, while constant agitation of the solution was insured by a magnetic stirrer.

At given irradiation time intervals, MB aliquots (5 mL) are taken out and the ratio of the final and initial MB dye solution concentration is analyzed, by UV–Vis spectrophotometer (ZUZI 4201/20) via using the maximum absorbance of MB at 665 nm based on the following formula:

(1) $$Degradation=(1-C/C_{0})\times 100\%$$

where $C_{0}$ and *C* represents the concentration of dye solution before and after irradiation, respectively.

## Figures and Tables

**Table 1:** Compressive and flexural strength of cement mortars containing nano-TiO_2_ sols annealed up to 400 °C.

		**Sample ID**	**2 days**	**7 days**	**28 days**	**90 days**	**Improvement (%)**
Compressive strength (MPa)		OPC	26.09	45.43	56.00	55.91	
		WR01	29.51	43.47	52.12	55.68	−0.4%
		WR02	29.11	42.08	52.94	57.92	3.6%
		WR05	28.04	44.88	49.36	54.71	−2.1%
		WR1	27.00	41.16	49.98	55.38	−0.9%
		NR01	29.75	45.51	53.94	55.18	−1.3%
		NR02	27.50	41.84	51.27	61.24	9.5%
		NR05	29.43	42.40	55.56	61.59	10.2%
		NR1	28.54	42.78	53.24	62.26	11.4%
		**Sample ID**	**2 days**	**7 days**	**28 days**	**90 days**	**Improvement (%)**
Flexural strength (MPa)		OPC	5.43	7.10	8.04	7.64	
		WR01	5.18	4.87	6.51	8.31	8.7%
		WR02	4.27	6.22	6.09	8.69	13.7%
		WR05	5.15	6.15	6.61	7.56	−1.0%
		WR1	4.71	6.28	5.92	8.03	5.1%
		NR01	4.86	6.13	7.99	8.30	8.6%
		NR02	5.00	5.61	5.19	7.66	0.2%
		NR05	5.13	5.60	6.36	7.46	−2.4%
		NR1	5.06	5.43	5.79	7.94	3.9%

**Table 2:** Summary of experimental conditions for the samples prepared.

**TiO_2_ sols**						
**Sample ID**	**Annealing Temperature °C**		**Heating (min)**	**Stabilization (min)**	**Cooling (min)**	
Control	-		-	-	-	
100TiO_2_	100		60	10	8	
200TiO_2_	200		140	2	8	
300TiO_2_	300		220	2	8	
400TiO_2_	400		300	2	8	
**nano-TiO_2_ mortar**						
**Mixture ID**	**Water (mL)**	**Cement (g)**	**Sand (g)**	**nano-TiO_2_ (g)**	**Curing (h)**	**Hydration (days)**
OPC	225	450	1350	0	24	2, 7, 28, 90
NR01	225	450	1350	0.45	24	2, 7, 28, 90
NR02	225	450	1350	0.9	24	2, 7, 28, 90
NR05	225	450	1350	2.25	24	2, 7, 28, 90
NR1	225	450	1350	4.5	24	2, 7, 28, 90
WR01	225	449.55	1350	0.45	24	2, 7, 28, 90
WR02	225	449.1	1350	0.9	24	2, 7, 28, 90
WR05	225	447.75	1350	2.25	24	2, 7, 28, 90
WR1	225	445.5	1350	4.5	24	2, 7, 28, 90

NR: no cement replacement, WR: with cement replacement

**Table 3:** Mix proportion of the specimens prepared for photocatalysis studies.

Mixture ID	Water (mL)	Cement (g)	Sand (g)	nano-TiO_2_ (g)	Hydration (days)
Control	225	450	1350	0	2
M01	225	450	1350	0.45	2
M02	225	450	1350	0.9	2
M05	225	450	1350	2.25	2
M1	225	450	1350	4.5	2
